# Oyster Aquaculture Impacts on Environment and Microbial Taxa in Dapeng Cove

**DOI:** 10.3390/microorganisms13112480

**Published:** 2025-10-30

**Authors:** Fei Tong, Xue Feng, Huarong Yuan, Yuxiang Chen, Pimao Chen

**Affiliations:** 1 South China Sea Fisheries Research Institute, Chinese Academy of Fishery Sciences/Scientific Observing and Experimental Station of South China Sea Fishery Resources and Environments, Ministry of Agriculture and Rural Affairs/Key Laboratory of Marine Ranching, Ministry of Agriculture and Rural Affairs, Guangzhou 510300, China; fengxue@scsfri.ac.cn (X.F.); yhr@scsfri.ac.cn (H.Y.); chenyuxiang@scsfri.ac.cn (Y.C.); chenpm@scsfri.ac.cn (P.C.); 2 Southern Marine Science and Engineering Guangdong Laboratory (Zhuhai), Zhuhai 519000, China

**Keywords:** microbial ecology, environmental microbiology, disturbances, oyster aquaculture, metagenomics

## Abstract

Environmental physicochemical factors and microorganisms play critical roles in the health of oysters. However, the impact of high-density oyster farming—a highly efficient filter-feeding bivalve system—on environmental conditions and microbial community structure and function remains poorly understood. This study conducted four-season monitoring of the water and sediment parameters in a semi-enclosed bay commercial oyster aquaculture (OA) system and a control area (CT), coupled with 16S rRNA amplicon sequencing of the environmental microbiota. Oyster aquaculture caused negligible disruption to water column parameters but significantly increased the concentrations of total organic carbon (TOC, annual mean OA vs. CT:1.15% vs. 0.56%), sulfides (annual mean OA vs. CT:67.72 vs. 24.99 mg·kg^−1^), and heavy metals (Cd, Pb, Cu, Zn, and Cr) in the sediment. α-diversity (Shannon and Chao indices) exhibited minimal overall perturbation, with significant inter-regional differences observed only in winter for both water and sediment. The bacterial community structure of the water column was significantly altered only in winter, whereas sediment communities showed structural shifts in spring, summer, and autumn. Water microbiota were primarily influenced by turbidity, dissolved oxygen, salinity, the Si/N ratio, and silicates. Sediment microbiota were correlated with Pb, Cu, Zn, TOC, Cr, and sediment particle size. Water bacterial functions displayed only four significantly divergent biogeochemical processes annually (sulfur compound respiration; OA vs. CT). In contrast, sediment bacteria exhibited 29 significantly disrupted functions annually, with the greatest seasonal divergence in winter (11/67 functions). Spring, summer, and autumn sediment functions showed distinct patterns. Understanding these environmental–microbial interactions is essential for sustainable oyster aquaculture and ecological optimization.

## 1. Introduction

Oysters can improve the water quality by removing plankton in the water column through filter-feeding [1]. Oysters thus provide high-quality protein for human consumption as well as ecosystem services [2]. In recent years, there have been increasing studies on the interaction between oyster aquaculture and environmental ecology [3,4]. Oysters are generally cultured using coastal bay rafts. In high-density culture areas, excess shellfish excreta results in the deposition of organic matter at the bottom of the water column [5]. The accumulation of this organic matter increases the decomposition activity of microorganisms and increases the dissolved oxygen (DO) consumption rate of sediments [6]. When the accumulation of sediments reaches a certain level beyond the maximum capacity of the culture environment, total nitrogen (TN) will accumulate, producing an anaerobic environment [7,8].

Organic and inorganic nitrogen released by shellfish feces and pseudofeces causes self-pollution in aquaculture systems [9]. In pursuit of higher profits, farmers tend to increase the density of inshore cultures, resulting in increased oyster waste, and raft culture devices alter the water flow velocity and direction, resulting in slower water exchange and material circulation [10,11]. This can lead to a decrease in the self-purification capacity of the seawater, and the biological sediments and excess nutrients deposited in the bay cannot be efficiently removed; this may lead to the deterioration of the environment in nearby waters, with increases in pathogenic microorganisms such as *Vibrio* sp. [12,13,14]. However, dissolved inorganic nitrogen (DIN), soluble reactive phosphorus (SRP), and other nutrients are primarily produced from the aerobic decomposition of excreta and organic substances, resulting in excessive nitrogen and phosphorus and eventual eutrophication of the water [7,15,16]. Previous studies have confirmed that waste products, such as residual bait and feces, produced by high-density aquaculture may influence the occurrence of offshore algal blooms [17].

In additionaddition, the filter-feeding of bivalves has a profound impact on the dynamics of the micro-food web [18]. The composition and abundance of microalgae directly affect the growth of filter-feeding shellfish [16]. Microbial taxa are closely related to the growth, breeding, and survival of oysters. For example, physicochemical factors such as the water temperature and dissolved oxygen (DO) significantly drive changes in the composition of marine microbial communities, and the aquaculture of cyanobacteria can increase oyster mortality. Therefore, marine bacterial communities directly affect oyster growth and survival [19,20]. During the development of the core microbial taxa of Pacific oyster spat, the microbial taxa in the spat are exchanged with the surrounding water environment, and the water microbiota will affect the survival rate of oyster spat [12,21,22]. Oysters may have specific selection mechanisms that allow a small number of beneficial microbial taxa to enter the tissue, while most of the oyster microbiota is controlled by environmental factors [23].

Oysters are closely linked to the dynamic fluctuation in environmental microorganisms [24]. However, it is not clear what the role and status of environmental microorganisms in oyster aquaculture systems in semi-enclosed urban bays are, as well as whether the high filtration efficiency of oysters has a long-term effect on the microbial community structure and function. Therefore, this study utilized high-throughput sequencing technology to analyze the changes in environmental microbiology functions across four seasons in oyster aquaculture areas in a semi-closed urban bay. Combined with the physical and chemical characteristics of the water column and sediments in the sea area, the impact of oyster aquaculture on the bacterial community structure was explored, providing a reference basis for the assessment of the potential impact of oyster aquaculture on the ecosystem and aquaculture management strategies.

## 2. Materials and Methods

### 2.1. Study Area

**Figure 1:**
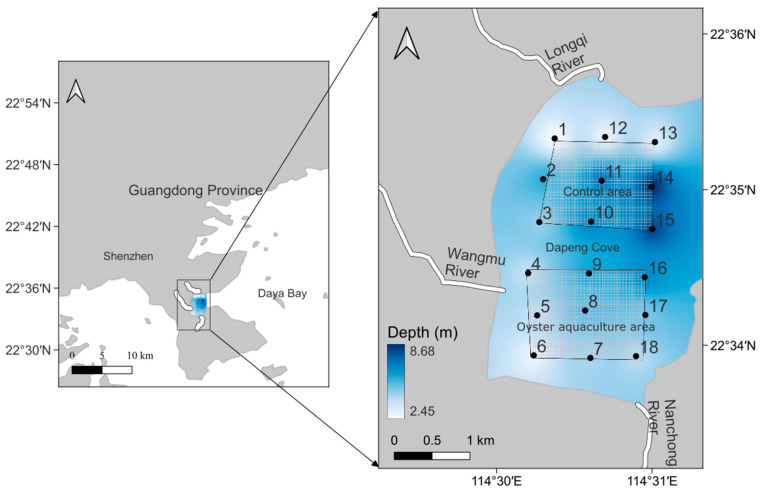
The sampling sites. Note: The numbers 1–18 in the figure represent the survey station numbers.

This study was carried out in the Dapeng cove in the eastern part of Shenzhen. The study area was a semi-enclosed bay with an area of approximately 10 km^2^. The area is affected by the subtropical monsoon, with two types of seasonal circulation: cyclonic circulation in summer and anticyclonic circulation in autumn, winter, and spring [25]. The annual average tidal range is 0.49 m, and the water velocity in the sea area is low (2~6 cm·s^−1^). To study the effect of oyster aquaculture on the microbial community, nine survey stations with *Crassostrea angulata* aquaculture rafts in the south of the sea area were set as the oyster aquaculture area (OA), and nine stations in the north were set as the control (CT) areas (Figure 1). The raft-culture area spans approximately 2 km^2^, with a biannual cycle for oyster cultivation on suspended ropes. Oyster spat are released in summer and harvested in winter. Each oyster shell typically hosts 10–20 spat, while each rope (3 m in length) holds approximately 20 shells at intervals of 30 cm. Recent annual production has reached 6.6 × 10^4^ tonnes, accounting for roughly 20% of the surveyed bay area.

### 2.2. Sample Collection and Processing

The study lasted from November 2020 to August 2021, and four sampling trips were conducted in spring (May), summer (August), autumn (November), and winter (February). The 18 survey stations evenly covered the entire area of Dapeng Cove. The spacing of sampling points was about 600 m. Water samples were collected using a Niskin sampler (Tianjin test center, Tianjin, China). Because the water depth in the bay is less than 10 m (average 5.2 m), surface water samples were taken at each sampling site. threeT 1 L water samples from each station were taken and mixed. After sample collection, about 1000 mL of the water sample was filtered through a 0.22 μm pore size Whatman GF/F filter (Whatman International Ltd., Maidstone, U.K.) with pre-acid cleaning and pre-combustion (500 °C, 5 h). The filter membrane was immediately stored in a liquid nitrogen tank for high-throughput sequencing. The filtrate was collected in a 500 mL acid-pre-cleaned polyethylene bottle, stored at a low temperature, and transported back to the laboratory within 24 h for nutrient analysis.

The concentration of chlorophyll a (Chl a) was measured using a portable chlorophyll a fluorescence tester (Aquafluor, Turner Designs, San Jose, CA, USA). Surface sea temperature (SST), Bottom bsea temperature (BST), salinity, and depth were measured using a portable CTD (CastAway, YSI, Yellow Springs, OH, USA). Turbidity (Tur) of the seawater was analyzed using a portable turbidity meter (WZB-175, Thunder Magnet, Shanghai, CN). The pH and DO levels were measured using a portable water quality analyzer (6600EDS, YSI, Yellow Springs, OH, USA). According to the ‘Marine “Monitoring Specification Part 4: Seawater Analysis’” (GB 17378.4-2007) and ‘Marine “Survey Specification Part 4: Seawater Chemical Elements Survey’” (GBT 12763.4-2007) [23], ammonium nitrogen (NH_4_^+^), nitrate nitrogen (NO_3_^−^), nitrite nitrogen (NO_2_^−^), silicate (SiO_3_^−^), chemical oxygen demand (COD), and SRP were measured using a spectrophotometer.

Surface sediment samples (0–10 cm) were mixed in triplicate from each station using a grab sediment sampler (Van Veen, OSIL, Hants, UK). The sediment samples were sieved with a 2 mm net to remove stones and plant roots, and then sealed in sterile plastic bags [26]. Total organic carbon (TOC) in the sediment was measured by the potassium dichromate oxidation–reduction volumetric method; total nitrogen (TN) was assessed using Kjeldahl titration; and total phosphorus (TP) was determined by spectrophotometry. Sulfide (Sf) analysis was conducted using methylene blue spectrophotometry. The sediments were analyzed for heavy metals (Cu, Pb, Zn, As, Hg, and Cd) using an atomic absorption spectrophotometer (Z-2010, Hitachi, Tokyo, Japan). The determination of As and Hg was carried out using atomic fluorescence spectrophotometry (AFS-8520, Beijing Haiguang, Beijing, China). Sediment particle size (Sps) was measured by sieve analysis. Sediment samples were gathered, preserved, transported, and tested following the Marine Monitoring Code Part 5: Sediment Analysis (GB 17378.5-2007) [23].

### 2.3. PCR Amplification and High-Throughput MiSeq Sequencing

In this study, the modified CTAB method was used to extract total genomic DNA from water and sediment samples [26]. DNA concentration and purity were determined by 1% (*w/v*) agarose gel. The DNA was diluted to 1 ng μL^−1^ with sterile water according to the concentration. Amplification of the V3-V4 variable region of the 16S rDNA genewas performed with a barcode-specific primer (338F-806R) [27]. All PCR reactions were made into a 20 μL reaction system using a kit (High-Fidelity PCR Master Mix, BioLab, Leading UK), including 0.8 μL of upstream and downstream primers and approximately 10 ng of template DNA. The reaction conditions were set with reference to Fang et al. [28]. After the reaction, electrophoresis was performed using 2% (*w/v*) agarose gels. The PCR products were combined in equal proportions and were then purified using a gel recovery kit (Qiagen, Hilden, Germany) [29]. DNA was quantified using a Quantus^TM^ fluorometer (Promega, Madison, WI, USA). The purified PCR products were sent for sequencing, and the DNA library was sequenced on the Illumina MiSeq platform to obtain 250 bp double-ended sequences.

### 2.4. Data Processing and Statistical Analysis

The map was created using QGIS 3.20 (QGIS Development Team, 2021 QGIS Geographic Information System). QIIME (V1.9.1) software was used to analyze the original sequence data. Any sequences shorter than 200 bp were removed, along with those having a quality control score lower than 20 or mismatches in barcodes or primers. UPARSE 7.0 software was used to cluster the sequences into Operational Taxonomic Units (OTUs) using a similarity criterion of 97%, and UCHIME was used to identify and delete chimeric sequences. RDP (V2.13) was used to classify and annotate the sequences of each sample based on the SILVA (V138) database. For downstream analysis, OTUs with ≥5 sequences in at least three samples were retained, and OTUs aligned to chloroplast and mitochondrial sequences were removed.

The alpha diversity (Shannon, Simpson, Chao 1, and Shannon evenness indices) of the samples was calculated, and the differences between the water column and sediment in different seasons were analyzed. Permutational multivariate analysis of variance (PERMANOVA) and analysis of similarity (ANOSIM) were used to analyze the spatial and temporal differences in the microbial community structure. The similarity of the community was visually analyzed by non-metric multidimensional scaling (NMDS). The Kruskal–Wallis (KW) test was used to compare group differences, and the Tukey–Kramer test was used for post hoc tests. A difference at the *p* < 0.05 level was considered significant. The data were expressed as mean ± standard deviation.

Redundancy analysis (RDA) was used to evaluate the relationship between the bacterial community and environmental factors. The variance inflation factor (VIF) was used to detect collinearity between variables (VIF greater than ten means significant collinearity). In the water column, the N/P ratio was deleted, and no collinearity index was detected in the sediment. The analysis was performed using the R (V4.2.1) vegan package. The above operations were based on the Meiji cloud platform (https://www.majorbio.com, accessed on 5 January 2025). FAPROTAX was used to predict the biochemical processes involving carbon (C), nitrogen (N), and sulfur (S) cycles of microorganisms in the water and sediment, and a difference analysis histogram was drawn based on Wilcoxon rank sum test statistics.

## 3. Results

### 3.1. Changes Physicalin and Chemical Factors

To compare the differences in environmental factors between the oyster culture area and the control area, the Mann-Whitney test was used. The analysis (Table 1) showed that only turbidity was significantly higher than that in the CT, and there were no significant differences in other water environmental factors (SST, salinity, NH_4_^+^, NO_3_^−^, DIN, SPR, pH, Chl a, and DO) between the two areas. The contents of TOC, Sf, Cd, Pb, Cu, Zn, and Cr in the sediments of OA were significantly higher than those in the CT (*p* < 0.05, Table 2), while particle size was significantly lower than that in the CT.

The seasonal differences in environmental factors in the two regions also fluctuated, and there were significant differences in SST, salinity, pH, Chl a, and DO in spring, SRP and Si/P in summer, Si/P in autumn, and SST, salinity, and Tur in winter (*p* < 0.05). In comparison, TOC, Pb, Cu, and Zn in the sediments of oyster aquaculture areas were significantly higher than those in the CT in each season (*p* < 0.05).

### 3.2. Characteristics of Microbial Taxa

**Figure 2:**
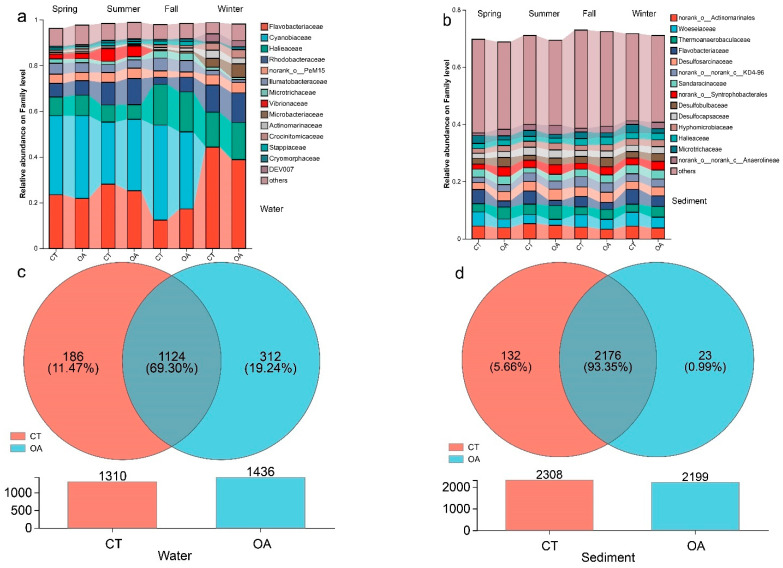
Species composition analysis diagram between oyster aquaculture and control areas. (**a**) The histogram of bacterial community composition in water column (Family level); (**b**) The histogram of bacterial community composition in sediment histogram (Family level); (**c**) The Venn diagrams and differential abundance of OUT in water column (OUT level); (**d**) The Venn diagrams and differential abundance of OUT in sediment (OUT level).

Flavobacteriaceae, Cyanobacteriaceae, Hariaceae, and Rhodobacteraceae were the dominant families in the water column (Figure 2a), accounting for more than 60% of the total family groups. The distribution of OTUs is shown in Figure 2c. The mean number of OTUs in the OA (1436) was higher than that in the CT (1310). The number of OTUs shared by the two regions was 1124, accounting for 69.30% of the total OTUs.

Desulfosarcinaceae, Flavobacteriaceae, Thermoanaerobaculaceae, Woeseiaceae, Microtrichaceae, and norank_o_Actinomarinales were the dominant families in the sediments (Figure 2b). The average number of OTUs in the sediments of the OA (2199) was lower than that of the CT (2308), and the number of OTUs shared by the two areas was 2176 (Figure 2d), accounting for 93.35% of the total OTUs.

The similarity in the bacterial community composition between OA and CT across the four seasons is shown in a heatmap (Figure S1). The sediments from the four seasons were clustered into one group, indicating high similarity in the bacterial community composition in OA in the four seasons. However, the composition in the water column in the four seasons did not cluster into one group, indicating that the bacterial community composition of the water body in the OA in each season was similar to that in the CT.

### 3.3. Microbial Community Structure Characteristics

There was no significant difference in the annual α diversity of the bacterial community structure in the water column between the OA and CT (Wilcoxon rank sum test, *p* > 0.05), and there was no significant difference in the annual α diversity of the sediment bacterial community in the two areas (*p* > 0.05). For the analysis by season, there was no significant difference in sediment α diversity between the two regions across the four seasons. However, there were significant differences in the water column between the two regions in winter (Table 3). The Shannon diversity and Chao species richness in the oyster breeding area were significantly different (*p* < 0.05), but the difference between the Simpson and Shannon evenness indices was not significant (Table 3).

**Figure 3:**
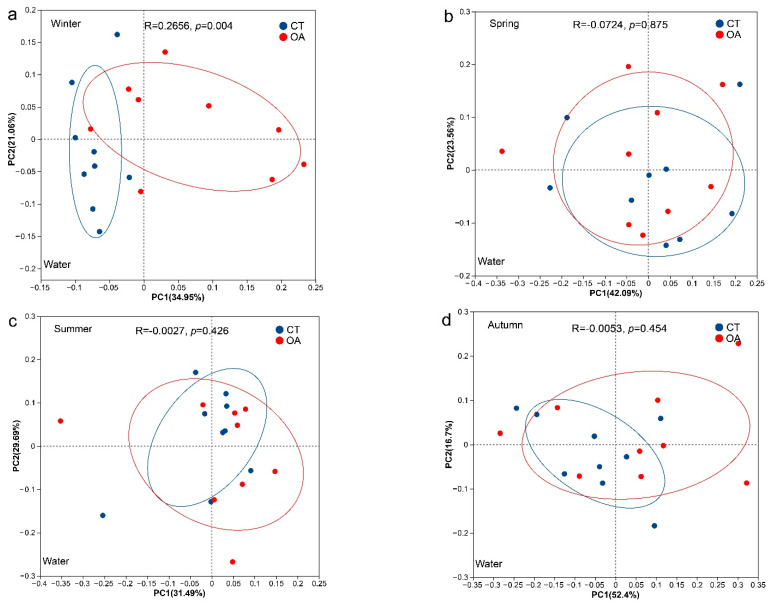

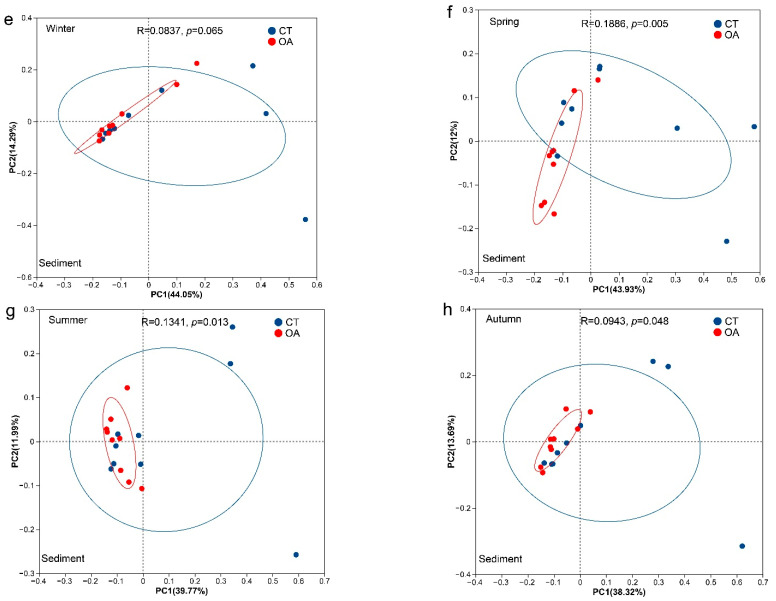
PCoA analysis of the bacterial community between the oyster aquaculture and control areas. (**a**–**d**) represent PCoA analysis of the bacterial community in the water column in winter, spring, summer and autumn respectively. (**e**–**h**) represent PCoA analysis of the bacterial community in the sediment in winter, spring, summer and autumn respectively. Notes: The ellipse indicates the 95% confidence ellipse.

The results of the PCoA analysis based on Bray–Curtis distance (OTU level) indicated no significant difference in the microbial community structure of the water between the OA and CT for the whole year (Figure 3b–d), except in winter (ANOSIM, R = 0.26, *p* = 0.004; Figure 3a). There were significant differences in the water column (ANOSIM, R = 0.14, *p* = 0.014; Figure S2a) and sediment (ANOSIM, R = 0.17, *p* < 0.001; Figure S2b) community structure between the annual OA and CT. In addition, there were also significant differences in the microbial community structure of sediments in spring, summer, and autumn (ANOSIM, *p* < 0.05; Figure 3f–h).

### 3.4. Differences in the Bacterial Community Structure and Species Composition

**Figure 4:**
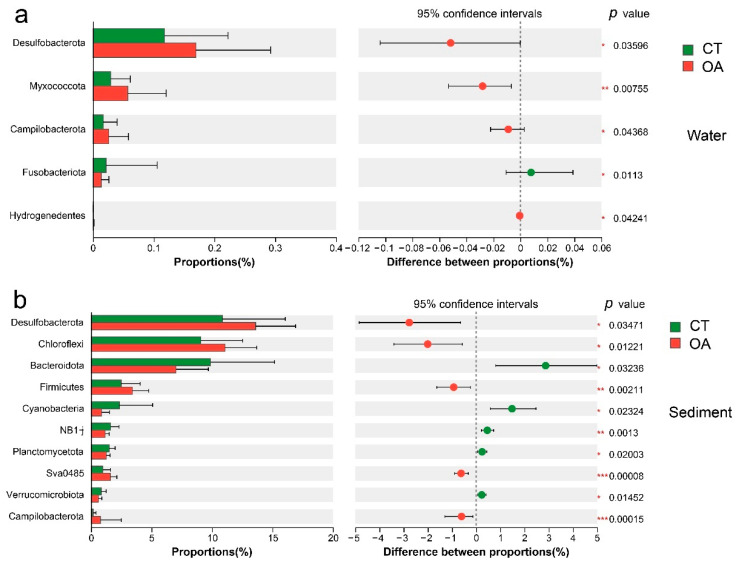
Bar plot for the average annual of bacterial relative abundance between oyster aquaculture and control areas (phylum level). (**a**) Bar plot for the average annual of bacterial relative abundance between oyster aquaculture and control areas (phylum level) in the water column. (**b**) Bar plot for the average annual of bacterial relative abundance between oyster aquaculture and control areas (phylum level) in the sediment. Note: * represents significant differences (* *p* < 0.05, ** *p* < 0.01, *** *p* < 0.001).

At the phylum level, the differences in the relative abundance of bacteria in the water column in summer and autumn were not significant. Only Schekmanbacteria were significantly different in spring (Wilcoxon rank sum test, *p* < 0.05; Figure S3a), and 10 phyla had significant differences in winter (*p* < 0.05; Figure S3b). There were significant differences in the average annual bacterial abundance of the five phyla, Desulfobacterota, Myxococcota, Campilobacterota, Fusobacteriota, and Hydrogenedentes (*p* < 0.05) (Figure 4a).

There were significant differences in the relative abundance of 22 phyla in the sediments of the two regions in spring, summer, autumn, and winter (*p* < 0.05). Among these, there were significant differences between the two regions in the relative abundance of 15, 3, 2, and 8 phyla during spring, summer, autumn, and winter, respectively. In spring, the relative abundance of Desulfobacterota, Chloroflexi, and Gemmatimonadota in oyster farms was significantly higher (*p* < 0.05) than that in the CT, while the relative abundance of Bacteroidota and Planctomycetota in oyster farms was significantly lower than that in the CT (Figure S3c). In summer, Sva0485, Deinococcota, and FCPU426 in the oyster farming area had significant differences (Figure S3d). In the autumn, the abundances of Caldisericota in oyster farms were significantly higher (*p* < 0.05) than those in the CT, while the relative abundances of FCPU426 in oyster farms were significantly lower (*p* < 0.05) than in the CT (Figure S3e). In the winter, the abundances of Firmicutes, Campilobacterota, NKB15, and Caldisericota in oyster farms were significantly higher (*p* < 0.05) than those in the CT, while the relative abundances of Planctomycetota, NB1-j, Verrucomicrobiota, and RCP2-54 in oyster farms were significantly lower (*p* < 0.05) than in the CT (Figure S3f). Overall, the average annual relative abundances of Desulfobacterota, Chloroflexi, Firmicutes, and Campilobacterota of oyster farms were significantly higher (*p* < 0.05) than the CT, while the relative abundances of Bacteroidota, Cyanobacteria, NB1J, Planctomycetota, and Verrucomicrobiota in oyster farms were significantly lower (*p* < 0.05) than in the CT (Figure 4b).

### 3.5. Environmental Driving Factors of Microbial Community Structure Difference

**Figure 5:**
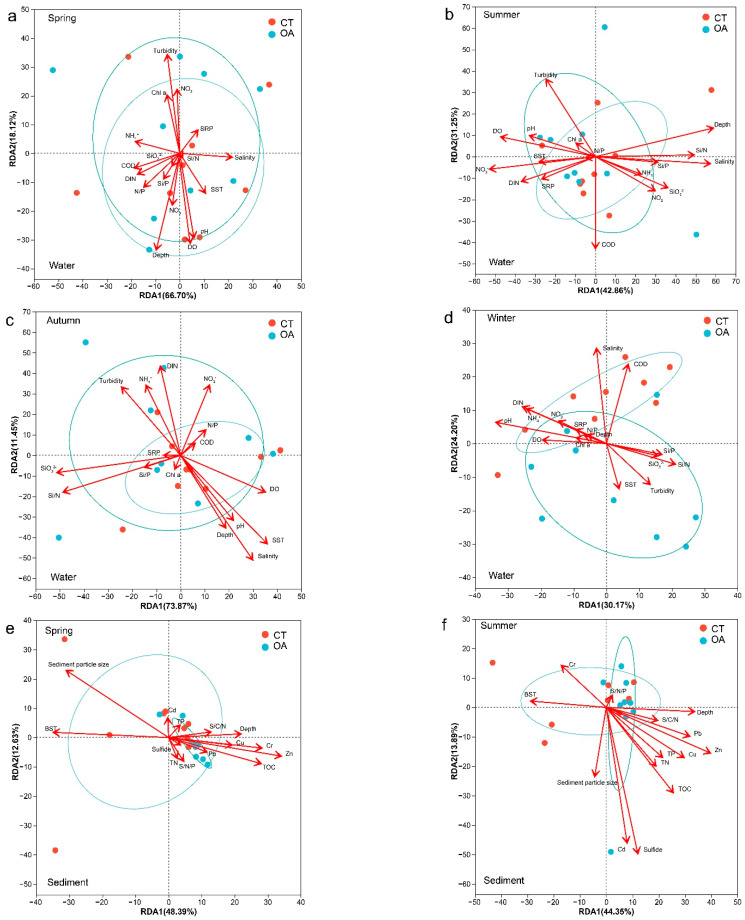

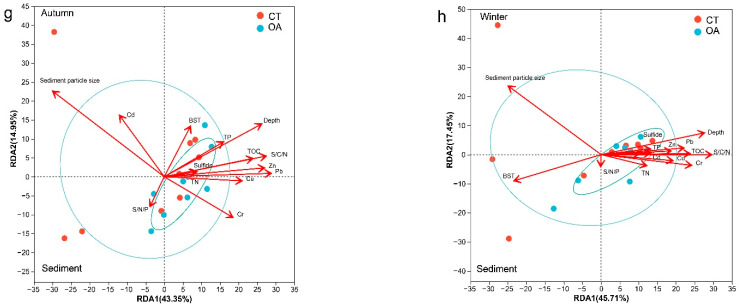
RDA for the oyster aquaculture and control areas bacterial communities and environmental factors (OTU level). (**a**–**d**) represent RDA for the oyster aquaculture and control areas bacterial communities and environmental factors (OTU level) in the water column in spring, summer autumn and winter respectively. (**e**–**h**) represent RDA for the oyster aquaculture and control areas bacterial communities and environmental factors (OTU level) in the sediment in spring, summer autumn and winter respectively. Notes: The ellipse indicates the 95% confidence ellipse.

The RDA revealed the relationships between the environmental factors and bacterial community structural characteristics (Figure 5). To better understand the direct relationship between environmental factors and community structure, Pearson correlation was used to test the collinearity between environmental factors and the bacterial community structure. A permutation test was used to calculate the significance level of environmental factors affecting the bacterial community structure. The first two axes of the investigated water environmental factors at the OTU level explain 84.82% (spring), 74.11% (summer), 85.32% (autumn), and 47.78% (winter) of the total variance. Overall, the average annual abundance of bacterial communities was significantly (*p* < 0.05; Figure S4a) correlated with all water environmental factors measured in this survey. However, there were differences among seasons. The environmental factors driving the fluctuation in bacterial community structure in the spring water column were turbidity (r = 0.52, *p* = 0.003), depth (r = 0.53, *p* = 0.004), DO (r = 0.43, *p* = 0.017), and pH (r = 0.39, *p* = 0.035) (Figure 5a). The main drivers of summer were depth (r = 0.31), salinity (r = 0.28), and NO_3_^−^ (r = 0.24) (Figure 5b). However, the correlations for these factors did not reach the specified significance level. The main drivers of autumn were SST (r = 0.42, *p* = 0.016), salinity (r = 0.46, *p* = 0.022), Si/N (r = 0.35, *p* = 0.049), and SiO_3_^2−^ (r = 0.35, *p* = 0.05) (Figure 5c). The main driver in winter was pH (r = 0.41, *p* = 0.016) (Figure 5d).

Overall, the average annual abundance of sedimental bacterial communities was significantly correlated with depth, Pb, Cu, Zn, TOC, Sps, and Cr (Figure S4b). The RDA revealed the relationships between the sedimental environment factors and bacterial community structure characteristics of the OA and CT. The first two axes of the investigated sedimental environment factors at the OTU level explained 61.02% (spring), 58.24% (summer), 58.30% (autumn), and 63.16% (winter) of the total variance. The environmental factors driving the fluctuation in the bacterial community structure in spring sediment were BST (r = 0.70, *p* = 0.001), Zn (r = 0.69, *p* = 0.001), Sps (r = 0.85, *p* = 0.003), TOC (r = 0.49, *p* = 0.005), and Cr (r = 0.46, *p* = 0.005) (Figure 5e). The main drivers in summer were Zn (r = 0.46, *p* = 0.008), Sf (r = 0.71, *p* = 0.009), Cd (r = 0.60, *p* = 0.022), and TOC (r = 0.39, *p* = 0.031) (Figure 5f). The main drivers in autumn were Pb (r = 0.49, *p* = 0.002), depth (r = 0.52, *p* = 0.005), Sps (r = 0.35, *p* = 0.049), Zn (r = 0.43, *p* = 0.008), C/N (r = 0.46, *p* = 0.009), and TOC (r = 0.35, *p* = 0.031) (Figure 5g). The main drivers in winter were depth (r = 0.57, *p* = 0.001), C/N (r = 0.62, *p* = 0.002), Sps (r = 0.85, *p* = 0.003), BST (r = 0.43, *p* = 0.003), Cr (r = 0.43, *p* = 0.007), and TOC (r = 0.40, *p* = 0.023) (Figure 5h).

### 3.6. Differences in Biogeochemical Functions of the Bacterial Community

**Figure 6:**
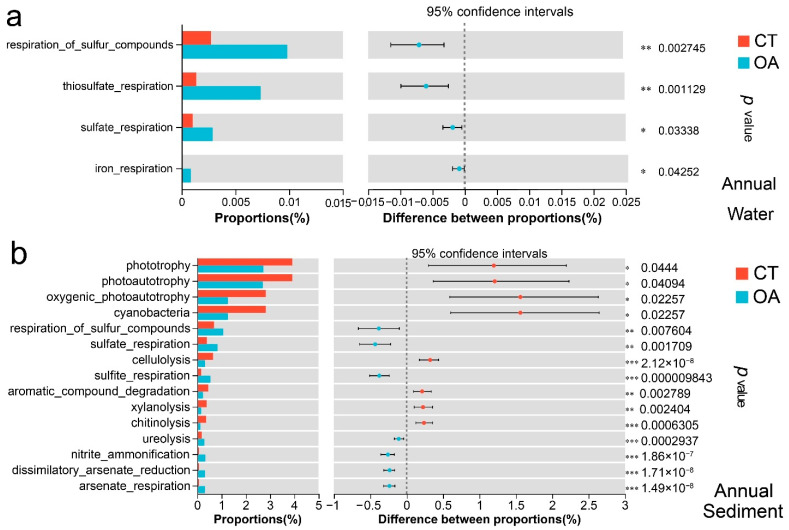
Analysis of potential biogeochemical functional differences in the bacterial community between oyster aquaculture and control areas. (**a**) Analysis of potential biogeochemical functional differences in the bacterial community between oyster aquaculture and control areas at annual level in the water column. (**b**) Analysis of potential biogeochemical functional differences in the bacterial community between oyster aquaculture and control areas at annual level in the sediment.Note: * represents significant differences (* *p* < 0.05, ** *p* < 0.01, *** *p* < 0.001).

According to the functional annotation obtained using FAPROTAX, a total of 73 biogeochemical cycle-related functions were compared with the water bacterial community in the CT over the course of a year. Four functions had significant differences between the two regions (Wilcoxon rank sum test, *p* < 0.05; Figure S5a). The functions of respiration of sulfur compounds, thiosulfate respiration, sulfate respiration, and iron respiration in the oyster aquaculture were significantly different from those in the CT. There was no difference in biochemical function between the two regions in spring or autumn. In summer, only dark sulfide oxidation, thiosulfate respiration, and manganese oxidation with low functional abundance were significantly different (Wilcoxon rank sum test, *p* < 0.05). There were differences in biogeochemical functions in the two regions in winter. Among 67 biogeochemical cycle-related functions that were compared with the water bacterial community in the CT in winter, the relative abundances of chemical energy heterotrophy and aerobic chemoheterotrophy were relatively high, accounting for 85% of the total predicted functional abundance. Among the 67 related functions, 11 demonstrated significant differences between the two regions (Wilcoxon rank sum test, *p* < 0.05) (Figure 6a). The CT exhibited significantly higher oxidative chemoheterotrophy, with dark hydrogen oxidation rates significantly higher than those of the OA. In contrast, the OA showed significantly higher rates of nitrate reduction, fermentation, sulfate respiration, dark oxidation of sulfur compounds, and thiosulfate respiration compared to the CT (*p* < 0.05).

The FAPROTAX functional annotation of 73 biogeochemical cycle-related functions that were compared between the sediment bacterial community in the OA and CT throughout the year showed that 29 were significantly different between the two regions (Wilcoxon rank sum test, *p* < 0.05).

Figure 6b shows the top 15 functions with significant differences in abundance. The functions of phototrophy, photoautotrophy, and cyanobacteria in the CT were significantly more abundant than those in the OA (*p* < 0.05), while the functions of nitrite ammonification, ureolysis, and sulfate respiration in the OA were significantly higher than those in the CT (*p* < 0.05).

The functional differences in the sediments of oyster aquacultures among the four seasons and the biogeochemical cycle in the CT are also shown. Spring included 16 differential functional groups, such as oxygenic photoautotrophy, cyanobacteria, and sulfate respiration (Figure S5b). In summer, there were 11 different functional groups, including cellulolysis, sulfite respiration, and nitrite ammonification (Figure S5c). Autumn demonstrated nine differential functional groups, including cellulolysis, aromatic compound degradation, and nitrite ammonification (Figure S5d). In winter, there were 10 significant functions, primarily in cellulolysis, sulfite respiration, and dissimilatory arsenate reduction (Figure S5e).

## 4. Discussion

The abundance and species composition of environmental microbiota are directly related to oyster diseases and the breeding environment [23]. In addition, microbial-mediated denitrification and sulfate reduction in the oyster aquaculture environment are closely related to the biogeochemical cycles in the habitat [30,31,32]. This study monitored the seasonal fluctuations in environmental factors and microbiota in oyster aquacultures and analyzed the drivers of bacterial community characteristics.

### 4.1. Oyster Aquaculture Disturbs Environmental Factors

Raft aquaculture of shellfish is typically established in marine areas with low wave activity and weak hydrodynamic conditions. High-density and intensive aquaculture practices generate residual feed and excretory waste that may alter environmental conditions in the surrounding waters [33]. Research by Rodhouse et al. [34] on mussels (*Mytilus edulis* L.) in Killary Harbour, Ireland, demonstrated that raft-cultured mussels annually released 8.5 kg (hm^−2^) of carbon and 1.1 kg (hm^−2^) of nitrogen into the marine environment. Similarly, mussel farming along the coasts of the Yellow Sea and Bohai Sea discharges approximately 101 tons of nitrogen and 15.5 tons of phosphorus annually [35]. NH_4_^+^ produced by direct oyster excretion and mineralization of biological sediments may release significant amounts of NO_3_^−^ into the water through oxidation [36]. However, our findings indicate no significant seasonal differences in NH_4_^+^, NO_3_^−^, SiO_3_^2−^, or COD between the OA and CT areas in Dapeng Cove Bay. This suggests that oyster raft aquaculture exerts minimal disturbance on nutrient concentrations in this semi-enclosed bay, with localized and minor impacts on environmental factors compared to other forms of aquaculture [37].

Our study revealed significantly higher SRP levels in oyster-enhanced areas during summer compared to control areas. This phenomenon may have multiple causes: (1) Elevated summer temperatures enhance oyster metabolism and sediment mineralization, thereby increasing SRP concentrations [38,39]. (2) Higher rainfall in summer elevates the phosphate flux from the nearby Wangmu River relative to the Longqi River in the control area [40]. Oyster raft farming not only affects nutrient dynamics but also alters the turbidity of the water [41,42]. Although there was no significant difference in turbidity in spring, summer, or autumn due to strong hydrodynamic conditions, there was a clear change in the turbidity of the water column in winter. The reduced water flow velocity in raft areas prolongs the residence time of suspended particulate matter, significantly increasing turbidity under weak hydrodynamic conditions [10,43].

The primary environmental impacts of shellfish aquaculture arise from biological sedimentation and the associated retention of nutrients [44]. Our data show that total organic carbon (TOC) in sediments from OA was significantly higher than in controls, attributable to fecal and pseudofecal deposits from oyster filtration. These results align with the findings by Xu et al. [45] during studies in the Maowei Sea, Guangxi, and further suggest that oyster-derived sedimentation may significantly influence marine carbon cycling. Sediment sulfide levels in farming areas were also elevated, consistent with prior studies [46,47,48]. Notably, sulfide concentrations decline rapidly after oyster aquaculture ceases [49], as microbial degradation preferentially utilizes C and N in organic particulates, leaving sulfides in the sediments [50].

Heavy metals such as Cd, Zn, and Cr were significantly enriched in oyster aquaculture sediments, mirroring the observations by Liu et al. [51] in Xiangshan Harbour for *Crassostrea plicatula*. Oysters exhibit strong bioaccumulation of heavy metals, but their tissue capacity is limited [52]. Heavy metals that exceed the enrichment capacity of oysters will be deposited together on the seabed through biological deposition. In addition, the filter-feeding and biological deposition of oysters may alter the particle size of sediments, resulting in a negative correlation between the content of heavy metals in sediments and particle size and a positive correlation with the content of organic matter [53]. Due to the high specific surface area and organic matter content of biological deposits such as feces, a large number of fine particles produced by filter-feeding shellfish may absorb more heavy metals [54]. Concurrently, elevated sulfides can alter the macrobenthic and microbial community structure, reducing bioturbation and metal enrichment by benthic organisms, thereby amplifying metal retention in sediments [15,55].

### 4.2. Effects on the Marine Microbial Community Structure

Microbial community dynamics serve as sensitive indicators of ecosystem health, reflecting environmental stressors and food web interactions across spatiotemporal scales [56]. As filter feeders, oysters selectively modify the plankton biomass and community structure [57], while nutrient shifts in farming areas further influence plankton assemblages [58].

The dominant microbial taxa in the water column showed minimal variation between the OA and CT areas, likely due to strong hydrodynamic exchange in Dapeng Cove. Although oyster-derived NH_4_^+^ and mineralized organic wastes can locally alter nutrient ratios and stimulate microbial shifts via “bottom-up” effects [59,60,61], such perturbations are spatially and temporally constrained. Tidal forces in spring, summer, and autumn rapidly disperse these nutrients [62,63], whereas the weaker rate of water exchange in winter produces differences in planktonic communities [63,64]. The RDA analysis showed that the water environment factors driving community structure fluctuations varied among the seasons. The turbidity, DO, pH, SST, salinity, and SiO_3_^2−^ of the water column had significant impacts on the fluctuations in the bacterial community structure among the seasons. There are environmental factors that had significant differences between the two regions but did not significantly affect the structure of the community. For example, SRP in summer and turbidity in winter had significant differences in the two regions, but these factors did not cause significant differences in the community structure of the water columns in the two regions. This also shows that the bacterial community structure of the water column has a certain level of stability [65].

Microorganisms buried in seabed sediments constitute an important part of the chemical cycle of the biosphere and play a key role in biogeochemical processes, including the carbon, nitrogen, and sulfur cycles. Environmental changes can be monitored by studying the microbial community, as it will quickly adapt to changes in the environment [66]. The results of community similarity analysis showed that, in contrast to the characteristics of the water community structure, the microbial community structure in the sediments of the OA and CT had significant differences among the seasons, except in winter. From top to bottom, oysters accumulated more nutrients in sediments through local deposition of feces and pseudofeces, and rich biological deposition could promote the precipitation of sulfides and nutrients into the sediments. These factors may be the cause of the changes in sediment microbial community structure [47]. The RDA analysis (Figure 5) revealed that the Sps and TOC were the primary drivers of the differences in sediment bacterial community structure between the OA and CT. In addition to the biodeposition by oysters that can directly increase the TOC content in the sea area, the biodeposition of oysters and oyster shell debris also affects the seabed sedimentary particle size spectrum. Marine deposition forms loose and porous sedimentary structures. Changes in these nutrients and the spatial structure of sediment particles are important factors driving the changes in the microbial community structure of oyster farm areas [16,47,60,67]. In addition, the RDA analysis indicated that the characteristics of the bacterial community in the sediment of the OA were affected by heavy metals. This may be due to the fact that the OA enriched the heavy metals in the sediments; as heavy metals are toxic and the tolerance of the bacterial community to heavy metals may differ between areas, this became one of the factors driving the structural changes in the sediment community of this area [68]. In winter, the metabolism and biodeposition of oysters decreased; the temperature decreased, and the abundances of opportunistic species and phytoplankton decreased, leading to the convergence of sediment bacterial communities in the OA and CT [14,45,66,69]. The RDA analysis of sediments in winter also showed that temperature was a key factor driving the community structure of the seabed. In turn, changes in the bacterial community structure may further affect the growth of oysters from the bottom up. For example, sulfate-reducing bacteria play an important role in the formation of the shells of the Pacific oyster (*Magallana gigas*), as they can affect the chemical composition of oyster shell carbonates [70].

### 4.3. Oyster Aquaculture Affects Biogeochemical Functions

Functional redundancy in microbial metabolic pathways reflects adaptive responses to geochemical variability [66]. FAPROTAX was used to examine the biogeochemical functions of the bacterial community in the survey area. The results of comparisons between the OA and CT areas showed that in the water environment, differences related to the N and S cycles, such as nitrate reduction and sulfate respiration, were observed in the two areas in winter and summer, consistent with the results for the community structure of bacterioplankton in the two areas. The direct discharge of N-, P-, and S-containing metabolites into the water column altered the biogeochemical cycles of the water column [60,61,71]. In contrast, the filtering effect of oysters altered the community structure of microorganisms and thereby affected the biogeochemical cycles mediated by microorganisms [72]. During the seasons with greater water exchange, the differences in biogeochemical functions of the two regions were not significant. In winters with weak hydrodynamic conditions and summers with strong biological metabolism, the differences in biogeochemical functions of the OA and CT were more pronounced [73]. In spring and autumn, there were no differences in the biogeochemical functions of oyster aquaculture and CT areas, a result that may be closely related to the hydrodynamic characteristics, breeding scale, and role of breeding methods in the oyster farm area and its adjacent waters [74,75].

The functional groups with differences in the sediment bacterial community in the two regions were more abundant than those in the water column. Although there was no significant difference in the microbial community structure of the sediments between the OA and CT in winter, 10 potential functional differences related to biogeochemistry were still observed in the two areas, suggesting that the microorganisms in the sedimentary environment had a high degree of functional redundancy. The results of this study show that the sediment biogeochemical functions of the two regions in the four seasons had significant differences. Yan et al. [50] found that oyster aquaculture significantly enriched sulfur and iron cycle-related microorganisms in sediments, consistent with our observation that the abundances of Desulfobacterota, Chloroflexi, Firmicutes, and Sva0485 in the OA were significantly higher than those in the CT. In addition, the sediments in the OA significantly improved the functions of nitrate reduction, nitric oxide reduction, and nitrogen fixation related to the N cycle, as well as the functions of sulfate respiration and sulfite respiration related to the S cycle. The differences in these biogeochemical functions of the bacterial community respond well to the changes in environmental factors such as TOC, sulfate, and heavy metals that are significantly enriched in the environment. The results also showed that oyster farming enhanced the removal of nitrogen in the water and the sedimentary environment by stimulating denitrification, effectively promoted the circulation of nutrients, and may have affected the greenhouse gas footprint [76]. In the CT, strong cellulolysis occurred in all four seasons. The cell walls of many species of microalgae contain a high proportion of cellulose. The filter-feeding of oysters consumes microalgae in the environment, while the microalgae in the CT naturally die and settle into the sediments, causing strong cellulolysis [77]. Overall, the raft culture of oysters may have biogeochemical functions similar to those of natural oyster reefs, suggesting that oysters have the potential to sustainably produce animal protein while restoring the marine environment [31].

## 5. Conclusions

This study assessed the disturbances to the water column and sediment dynamics induced by oyster aquaculture in Dapeng Cove through monitoring the shifts in the microbial community structure. This approach provides insights into the role and status of oyster aquaculture within the bay environment. Oyster aquaculture activities appear to increase the enrichment of total organic carbon (TOC), sulfide, and certain heavy metals in the seabed via biodeposition processes. The high-throughput sequencing results further revealed that oyster aquaculture significantly influences the structure of microbial communities in the adjacent waters. However, the extent and magnitude of this impact are modulated by composite factors, including seasonal physicochemical parameters and hydrodynamic conditions. Additionally, oyster aquaculture promoted the biogeochemical cycling of elements such as nitrogen (N) and sulfur (S) within the sea area, mediated by changes in the resident microbial communities.

These findings demonstrate that a single quarterly survey is insufficient to fully elucidate the complex interactions between oyster aquaculture areas and the surrounding environment. Monitoring and evaluation across different seasons are essential to develop a more comprehensive understanding of the environmental impacts associated with oyster aquaculture. Future research will integrate metagenomics and other advanced technologies to conduct high-resolution assessments, enabling a more precise characterization of oyster aquaculture’s influence on the bay ecosystem.

## Figures and Tables

**Table 1:** Water environmental factors between oyster aquaculture and control at Dapeng Cove.

Water Environment Factor	OA	CT	*p*-Value				
			Annual Mean	Spring	Summer	Autumn	Winter
SST (°C)	25.94 ± 3.34	26.04 ± 3.45	0.900	0.005 **	0.444	0.166	0.009 **
Salinity (PSU)	32.96 ± 1.00	33.03 ± 0.97	0.749	0.036 *	0.510	0.738	0.003 **
NH_4_^+^ (mg·L^−1^)	0.10 ± 0.04	0.10 ± 0.04	0.813	0.720	0.113	0.555	0.650
NO_3_^−^ (mg·L^−1^)	0.09 ± 0.02	0.09 ± 0.02	0.871	0.523	0.609	0.248	0.330
DIN (mg·L^−1^)	0.19 ± 0.04	0.18 ± 0.04	0.764	0.927	0.250	0.890	0.830
SPR (μg·L^−1^)	6.13 ± 2.34	6.53 ± 2.85	0.521	0.159	0.006 **	0.168	0.338
SiO_3_^2−^ (mg·L^−1^)	0.12 ± 0.06	0.11 ± 0.06	0.664	0.288	0.313	0.054	0.555
Si/P	25.29 ± 16.89	25.73 ± 18.83	0.916	0.105	0.032 *	0.030 *	0.478
COD (mg·L^−1^)	0.95 ± 0.43	0.98 ± 0.45	0.827	0.742	0.773	0.520	0.678
Chl a (μg·L^−1^)	0.87 ± 0.55	0.78 ± 0.62	0.529	0.001 **	0.826	0.653	0.329
DO (mg·L^−1^)	7.51 ± 0.76	7.63 ± 0.57	0.418	0.037 *	0.618	0.682	0.914
pH	8.34 ± 0.20	8.33 ± 0.08	0.753	0.039 *	0.405	0.780	0.800
Turbitity (NTU)	5.37 ± 2.12	4.44 ± 1.65	0.021 *	0.066	0.224	0.122	0.040 *

Note: * represents significant differences (* *p* < 0.05, ** *p* < 0.01).

**Table 2:** Sediment environmental factors between oyster aquaculture and control at Dapeng Cove.

Sediment Environment Factor	OA	CT	*p*-Value				
			Annual Mean	Spring	Summer	Autumn	Winter
TOC (%)	1.15 ± 0.44	0.56 ± 0.25	<0.001 ***	0.003 **	0.003 **	0.006 **	0.007 **
TN (%)	0.13 ± 0.06	0.11 ± 0.05	0.055	0.413	0.201	0.284	0.073
TP (%)	0.21 ± 0.06	0.19 ± 0.08	0.257	0.413	0.08	0.047 *	0.983
C/N	12.33 ± 12.33	8.43 ± 9.15	0.133	0.258	0.546	0.007 **	0.007 **
Sulfide (mg·kg^−1^)	67.72 ± 70.03	24.99 ± 20.65	<0.001 ***	0.22	0.052	0.092	0.109
Cd (mg·kg^−1^)	0.04 ± 0.04	0.03 ± 0.02	0.022 *	0.213	0.179	0.43	0.1
Pb (mg·kg^−1^)	44.54 ± 18.05	19.64 ± 9.08	<0.001 ***	0.021 *	<0.001 ***	<0.001 ***	<0.001 ***
Cu (mg·kg^−1^)	34.81 ± 15.54	8.16 ± 4.98	<0.001 ***	0.005 **	<0.001 ***	0.001 **	<0.001 ***
Zn (mg·kg^−1^)	83.32 ± 34.79	38.66 ± 20.08	<0.001 ***	0.003 **	0.001 **	0.002 **	0.012 *
Cr (mg·kg^−1^)	63.54 ± 21.41	40.79 ± 21.95	<0.001 ***	0.013 *	0.633	<0.001 ***	<0.001 ***
Sps (mm)	0.01 ± 0.01	0.02 ± 0.02	<0.001 ***	0.064	0.734	0.104	0.19

Note: * represents significant differences (* *p* < 0.05, ** *p* < 0.01, *** *p* < 0.001).

**Table 3:** The bacterial community of oyster aquaculture and control areas water alpha diversity in winter.

Diversity Index	Control	Oyster Aquaculture	*p*
Shannon	2.91 ± 0.12	3.14 ± 0.15	0.01
Chao	344 ± 43.01	458.65 ± 101.09	0.01
Invsimpson	9.77 ± 1.65	11.11 ± 1.67	0.13
Shannoneven	0.53 ± 0.02	0.55 ± 0.01	0.13

## Data Availability

Sequence data reported in this work have been deposited into the NCBI database under the accession number PRJNA1221659.

## Supplementary Materials

The following supporting information can be downloaded at: https://www.mdpi.com/article/10.3390/microorganisms13112480/s1, Figure S1: Hierarchical clustering heatmap of bacterial community compositions between oyster aquaculture and control areas across four (seasonsfamily level); Figure S2: PCoA analysis of the bacterial community between the oyster aquaculture and control areas; Figure S3. Bar plot for the average annual of bacterial relative abundance between oyster aquaculture and control areas; Figure S4. RDA of bacterial communities and environmental factors in the oyster aquaculture and control areas; Figure S5. Analysis of potential biogeochemical functional differences in the bacterial community between oyster aquaculture and control areas.

## References

[1] Gawde R.K., North E.W., Hood R.R., Long W., Wang H., Wilberg M.J. (2024). A high resolution hydrodynamic-biogeochemical-oyster-filtration model predicts that the presence of oysters (*Crassostrea virginica*) can improve, or reduce, water quality depending upon oyster abundance and location. Ecol. Model..

[2] Martínez-Baena F., Lanham B.S., McLeod I.M., Taylor M.D., McOrrie S., Luongo A., Bishop M.J. (2022). Remnant Oyster Reefs as Fish Habitat within the Estuarine Seascape. Mar. Environ. Res..

[3] Deng Y., Cao Y., Xu Y., Wen G., Su H., Hu X., Xu W., Jie L., Yu Z. (2023). Study on Purification Effect of Shellfish and Algae Coupling on Intensive Aquaculture Tailwater. South China Fish. Sci..

[4] Song X., Song J., Yan Q., Zhou J., Cai Z. (2021). Assembly of a Benthic Microbial Community in a Eutrophic Bay with a Long History of Oyster Culturing. Microorganisms.

[5] Jeong H., Araújo D.F., Ra K. (2024). Combined Copper Isotope and Elemental Signatures in Bivalves and Sediments from the Korean Coast: Applicability for Monitoring Anthropogenic Contamination. Mar. Pollut. Bull..

[6] Tan K., Liu X., Yan X., Huang L., Luo C., Tan K., Kwan K.Y. (2024). Performance of Fishery Carbon Sink of Oyster Aquaculture (Mainly *Crassostrea hongkongensis*) in Guangxi, China: A Long-Term (2003–2022) Analysis. Estuar. Coast. Shelf Sci..

[7] Xie L., Yang B., Xu J., Dan S.F., Ning Z., Zhou J., Kang Z., Lu D., Huang H. (2024). Effects of Intensive Oyster Farming on Nitrogen Speciation in Surface Sediments from a Typical Subtropical Mariculture Bay. Sci. Total Environ..

[8] Coffin M.R.S., Clements J.C., Comeau L.A., Guyondet T., Maillet M., Steeves L., Winterburn K., Babarro J.M.F., Mallet M.A., Haché R. (2021). The Killer within: Endogenous Bacteria Accelerate Oyster Mortality during Sustained Anoxia. Limnol. Oceanogr..

[9] Comeau L.A., Mallet A.L., Carver C.E., Guyondet T. (2014). Impact of High-Density Suspended Oyster Culture on Benthic Sediment Characteristics. Aquacult. Eng..

[10] Gaurier B., Germain G., Kervella Y., Davourie J., Cayocca F., Lesueur P. (2011). Experimental and Numerical Characterization of an Oyster Farm Impact on the Flow. Eur. J. Mech. B/Fluids.

[11] Gadeken K., Clemo W.C., Ballentine W., Dykstra S.L., Fung M., Hagemeyer A., Dorgan K.M., Dzwonkowski B. (2021). Transport of Biodeposits and Benthic Footprint around an Oyster Farm, Damariscotta Estuary, Maine. PeerJ.

[12] Whittington R.J., Buller N., Pathirana E., Dhand N.K., Hair S., Hick P.M., Paul-Pont I. (2024). Investigations of the Involvement of *Vibrio* Species with *Ostreid Herpesvirus-1* in Mass Mortality Events in the Pacific Oyster *Crassostrea gigas*. Aquaculture.

[13] Labrie M.S., Sundermeyer M.A., Howes B.L. (2022). Modelling the Spatial Distribution of Oyster (*Crassostrea virginica*) Biodeposits Settling from Suspended Aquaculture. Estuaries Coasts.

[14] Samperio-Ramos G., Vidal-Nieves C., García-Esquivel Z., Herzka S.Z., Sandoval-Gil J.M., Camacho-Ibar V.F. (2024). Environmental Influence on Feeding and Biodeposition Rates of Pacific Oysters (*Crassostrea gigas*) throughout Its Culture Cycle in a Coastal Lagoon with Upwelling Influence. Estuaries Coasts.

[15] Yan Q., Jia Z.P., Song J.T., Zhou J., Cai Z. (2023). Oyster Culture Changed the Phosphorus Speciation in Sediments through Biodeposition. Environ. Res..

[16] Nascimento V.S.d., Lapa K.R., de Miranda Gomes C.H.A., Gray M., da Silva G., Garbossa L.H.P., Suplicy F.M., de Melo C.M.R. (2022). Filtration and Biodeposition Rates of Crassostrea Oysters for Southern Brazilian Waters. Reg. Stud. Mar. Sci..

[17] Jiang Z., Du P., Liao Y., Liu Q., Chen Q., Shou L., Zeng J., Chen J. (2019). Oyster Farming Control on Phytoplankton Bloom Promoted by Thermal Discharge from a Power Plant in a Eutrophic, Semi-Enclosed Bay. Water Res..

[18] Okumura Y., Masuda Y., Matsutani M., Shiomoto A. (2023). Influence of Oyster and Seaweed Cultivation Facilities on Coastal Environment and Eukaryote Assemblages in Matsushima Bay, Northeastern Honshu, Japan. Front. Mar. Sci..

[19] Liu M., Li Q., Tan L., Wang L., Wu F., Li L., Zhang G. (2023). Host-Microbiota Interactions Play a Crucial Role in Oyster Adaptation to Rising Seawater Temperature in Summer. Environ. Res..

[20] Lameira Silva O.L., Veríssimo S.M.M., da Rosa A.M.B.P., Iguchi Y.B., Nunes E.d.S.C.d.L., Moraes C.M., Cordeiro C.A.M., Xavier D.d.A., Pinto A.S.O., Peixoto Joele M.R.S. (2020). Effect of environmental factors on microbiological quality of oyster farming in amazon estuaries. Aquac. Rep..

[21] Cho A., Finke J.F., Zhong K.X., Chan A.M., Saunders R., Schulze A., Warne S., Miller K.M., Suttle C.A. (2024). The Core Microbiome of Cultured Pacific Oyster Spat Is Affected by Age but Not Mortality. Microbiol. Spectr..

[22] Ricketts O.M.A., Isaac S.R., Lara R.A., Mendela T.S., Enzor L.A., Silver A.C. (2024). Elevated Temperature and Decreased Salinity Impacts on Exogenous Vibrio Parahaemolyticus Infection of Eastern Oyster, Crassostrea Virginica. Front. Microbiol..

[23] Liu M., Li Q., Xu W., Wang L., Wu F., Tan L., Li L., Zhang G. (2024). Characterization of Water Microbiota and Their Relationship with Resident Oysters during an Oyster Mortality Event. Microbiol. Spectr..

[24] Fang G., Yu H., Zhang Y., Liang J., Tang Y., Liang Z. (2023). Diversities and Shifts of Microbial Communities Associated with Farmed Oysters (*Crassostrea gigas*) and Their Surrounding Environments in Laoshan Bay Marine Ranching, China. Microorganisms.

[25] Rao Y.Y., Cai L.Z., Chen B.W., Chen X., Zheng L., Lin S. (2020). How Do Spatial and Environmental Factors Shape the Structure of a Coastal Macrobenthic Community and Meroplanktonic Larvae Cohort? Evidence from Daya Bay. Mar. Pollut. Bull..

[26] Pierangeli G.M.F., Domingues M.R., Choueri R.B., Hanisch W.S., Gregoracci G.B., Benassi R.F. (2022). Spatial Variation and Environmental Parameters Affecting the Abundant and Rare Communities of Bacteria and Archaea in the Sediments of Tropical Urban Reservoirs. Microb. Ecol..

[27] Lu J., Yao T., Yu G., Ye L. (2023). Adaptive response of triploid fujian oyster (*Crassostrea angulata*) to nanoplastic stress: Insights from physiological, metabolomic, and microbial community analyses. Chemosphere.

[28] Fang G., Yu H., Sheng H., Tang Y., Liang Z. (2021). Comparative Analysis of Microbial Communities between Water and Sediment in Laoshan Bay Marine Ranching with Varied Aquaculture Activities. Mar. Pollut. Bull..

[29] Rajeev M., Sushmitha T.J., Toleti S.R., Pandian S.K. (2020). Sediment-Associated Bacterial Community and Predictive Functionalities Are Influenced by Choice of 16S Ribosomal RNA Hypervariable Region(s): An Amplicon-Based Diversity Study. Genomics.

[30] Le Ray J., Bec B., Fiandrino A., Lagarde F., Cimiterra N., Raimbault P., Roques C., Rigaud S., Régis J., Mostajir B. (2023). Impact of anoxia and oyster mortality on nutrient and microbial planktonic components: A mesocosm study. Aquaculture.

[31] Ray N.E., Fulweiler R.W. (2021). Meta-Analysis of Oyster Impacts on Coastal Biogeochemistry. Nat. Sustain..

[32] Ray N.E., Hancock B., Brush M.J., Colden A., Cornwell J., Labrie M.S., Maguire T.J., Maxwell T., Rogers D., Stevick R.J. (2021). A Review of How We Assess Denitrification in Oyster Habitats and Proposed Guidelines for Future Studies. Limnol. Oceanogr. Methods.

[33] Zhuang M., Fu S., Yao T., Lu J., Jiang H., Wang Y., Hao Y., Ye L. (2024). Perkinsus Spp. Infections between Cultured and Wild Oysters *Crassostrea hongkongensis* and Saccostrea Mordax. South China Fish. Sci..

[34] Rodhouse P.G., Roden C.M., Hensey M.P., Ryan T.H. (1985). Production of Mussels, *Mytilus edulis*, in Suspended Culture and Estimates of Carbon and Nitrogen Flow: Killary Harbour, Ireland. J. Mar. Biol. Assoc. UK.

[35] Zheng L., Zhai W., Wang L., Huang T. (2020). Improving the Understanding of Central Bohai Sea Eutrophication Based on Wintertime Dissolved Inorganic Nutrient Budgets: Roles of North Yellow Sea Water Intrusion and Atmospheric Nitrogen Deposition. Environ. Pollut..

[36] Rose J.M., Bricker S.B., Tedesco M.A., Wikfors G.H. (2014). A Role for Shellfish Aquaculture in Coastal Nitrogen Management. Environ. Sci. Technol..

[37] Forrest B.M., Keeley N.B., Hopkins G.A., Webb S.C., Clement D.M. (2009). Bivalve aquaculture in estuaries: Review and synthesis of oyster cultivation effects. Aquaculture.

[38] Gudasz C., Bastviken D., Steger K., Premke K., Sobek S., Tranvik L.J. (2010). Temperature-controlled organic carbon mineralization in lake sediments. Nature.

[39] Bruhns T., Timm S., Feußner N., Engelhaupt S., Labrenz M., Wegner M., Sokolova I.M. (2023). Combined Effects of Temperature and Emersion-Immersion Cycles on Metabolism and Bioenergetics of the Pacific Oyster *Crassostrea (Magallana) gigas*. Mar. Environ. Res..

[40] Zhang Y., Mao W., Li R., Liu Y., Wang P., Zheng Z., Guan Y. (2022). Distribution Characteristics, Risk Assessment, and Quantitative Source Apportionment of Typical Contaminants (HMs, N, P, and TOC) in River Sediment under Rapid Urbanization: A Study Case of Shenzhen River, Pearl River Delta, China. Process Saf. Environ. Prot..

[41] Plutchak R., Major K., Cebrian J., Foster C.D., Miller M.-E.C., Anton A., Sheehan K.L., Heck K.L., Powers S.P. (2010). Impacts of Oyster Reef Restoration on Primary Productivity and Nutrient Dynamics in Tidal Creeks of the North Central Gulf of Mexico. Estuaries Coasts.

[42] Xie L., Xu J., Yang B., Yang B., Ning Z., Zhu D., Lu D., Kang Z., Zhou J., Huang H. (2025). Oyster Farming and Hydrodynamic Conditions Regulate Composition and Sources of Sedimentary Organic Matter in a Typical River-Estuary-Bay Continuum. J. Hydrol..

[43] Campbell M.D., Hall S.G. (2019). Hydrodynamic Effects on Oyster Aquaculture Systems: A Review. Rev. Aquacult..

[44] Todorov S.D., Carneiro K.O., Lipilkina T.A., Do H.-K., Miotto M., De Dea Lindner J., Chikindas M.L. (2024). Beneficial Microorganisms for the Health-Promoting in Oyster Aquaculture: Realistic Alternatives. Aquacult Int..

[45] Xu C., Yang B., Dan S.F., Zhang D., Liao R., Lu D., Li R., Ning Z., Peng S. (2020). Spatiotemporal Variations of Biogenic Elements and Sources of Sedimentary Organic Matter in the Largest Oyster Mariculture Bay (Maowei Sea), Southwest China. Sci. Total Environ..

[46] Lacoste É., Gaertner-Mazouni N. (2016). Nutrient Regeneration in the Water Column and at the Sediment–Water Interface in Pearl Oyster Culture (*Pinctada margaritifera*) in a Deep Atoll Lagoon (Ahe, French Polynesia). Estuar. Coast. Shelf S.

[47] Erler D.V., Welsh D.T., Bennet W.W., Meziane T., Hubas C., Nizzoli D., Ferguson A.J.P. (2017). The Impact of Suspended Oyster Farming on Nitrogen Cycling and Nitrous Oxide Production in a Sub-Tropical Australian Estuary. Estuar. Coast. Shelf Sci..

[48] Azandégbé A., Poly F., Andrieux-Loyer F., Kérouel R., Philippon X., Nicolas J.-L. (2012). Influence of Oyster Culture on Biogeochemistry and Bacterial Community Structure at the Sediment-Water Interface. Fems Microbiol. Ecol..

[49] Sim B.-R., Kim H.C., Kang S., Lee D.-I., Hong S., Lee S.H., Kim Y. (2020). Geochemical Indicators for the Recovery of Sediment Quality after the Abandonment of Oyster *Crassostrea gigas* Farming in South Korea. Korean, J. Fish. Aquat. Sci..

[50] Yan Q., Song J.T., Zhou J., Han Y., Cai Z. (2022). Biodeposition of Oysters in an Urbanized Bay Area Alleviates the Black-Malodorous Compounds in Sediments by Altering Microbial Sulfur and Iron Metabolism. Sci. Total Environ..

[51] Liu Q., Liao Y.B., Zhu J.H., Shi X., Shou L., Zeng J., Chen Q., Chen J. (2023). Influence of Biodeposition by Suspended Cultured Oyster on the Distributions of Trace Elements in Multiple Media in a Semi-Enclosed Bay of China. J. Hazard. Mater..

[52] Shulkin V.M., Presley B.J., Kavun V.I. (2003). Metal Concentrations in Mussel Crenomytilus Grayanus and Oyster *Crassostrea gigas* in Relation to Contamination of Ambient Sediments. Environ. Int..

[53] Liang J., Liu J., Xu G., Chen B. (2019). Distribution and Transport of Heavy Metals in Surface Sediments of the Zhejiang Nearshore Area, East China Sea: Sedimentary Environmental Effects. Mar. Pollut. Bull..

[54] Ping X.Y., Zhang H., Jiang Y.Z., Ling J., Sun P., Tang B. (2023). Sediment Properties and Benthic Fauna Associated with Stock Enhancement and Farming of Marine Bivalve Populations in Xiangshan Bay, China. Aquac. Res..

[55] Liao Y.B., Liu Q., Shou L., Tang Y., Liu Q., Zeng J., Chen Q., Yan X. (2022). The Impact of Suspended Oyster Farming on Macrobenthic Community in a Eutrophic, Semi-Enclosed Bay: Implications for Recovery Potential. Aquaculture.

[56] Cook L.S.J., Briscoe A.G., Fonseca V.G., Boenigk J., Woodward G., Bass D. (2025). Microbial, holobiont, and tree of life eDNA/eRNA for enhanced ecological assessment. Trends Microbiol..

[57] Lavrentyev P.J., Gardner W.S., Yang L. (2000). Effects of the Zebra Mussel on Nitrogen Dynamics and the Microbial Community at the Sediment-Water Interface. Aquat. Microb. Ecol..

[58] Liu W., Bao Y.L., Li K.J., Yang N., He P., He C., Liu J. (2024). The Diversity of Planktonic Bacteria Driven by Environmental Factors in Different Mariculture Areas in the East China Sea. Mar. Pollut. Bull..

[59] Vezzulli L., Stagnaro L., Grande C., Tassistro G., Canesi L., Pruzzo C. (2018). Comparative 16SrDNA Gene-Based Microbiota Profiles of the Pacific Oyster (*Crassostrea gigas*) and the Mediterranean Mussel (*Mytilus galloprovincialis*) from a Shellfish Farm (Ligurian Sea, Italy). Microb. Ecol..

[60] Patil M.P., Woo H.-E., Kim J.-O., Kim K. (2022). Field study on short-term changes in benthic environment and benthic microbial communities using pyrolyzed oyster shells. Sci. Total Environ..

[61] Auladell A., Barberán A., Logares R., Garcés E., Gasol J.M., Ferrera I. (2022). Seasonal Niche Differentiation among Closely Related Marine Bacteria. ISME J..

[62] Sun C.-C., Wang Y.-S., Wu M.-L., Dong J.-D., Wang Y.-T., Sun F.-L., Zhang Y.-Y. (2011). Seasonal Variation of Water Quality and Phytoplankton Response Patterns in Daya Bay, China. Int. J. Environ. Res. Public Health.

[63] Wang S., Wu F., Gong X., Liu H., Rao Y., Zhang S., Hou G., Huang H. (2025). Effects of Water Mass Dynamics on the Structure and Distribution of Fish Egg Community in Daya Bay. Mar. Environ. Res..

[64] Guo Z., Xiao Y., Liu Y., Wu P., Li C. (2023). Long-Term Variations of Biogenic Elements and Nutritional Status in Daya Bay, Northern South China Sea. J. Mar. Sci. Eng..

[65] Lee J., Kang S.-H., Yang E.J., Macdonald A.M., Joo H.M., Park J., Kim K., Lee G.S., Kim J.-H., Yoon J.-E. (2019). Latitudinal Distributions and Controls of Bacterial Community Composition during the Summer of 2017 in Western Arctic Surface Waters (from the Bering Strait to the Chukchi Borderland). Sci. Rep..

[66] Gao L., Zhao Y., Wang Z., Zhang Y., Ming J., Sun X., Ni S.-Q. (2024). Seasonal and Distance-Decay Patterns of Surface Sediments Microbial Nitrogen and Sulfur Cycling Linkage in the Eastern Coast of China. Mar. Pollut. Bull..

[67] Liang Y., Zhang Y., Zhou C., Li H., Kang X., Wang L., Song J., Jiao N. (2019). Cumulative Impact of Long-Term Intensive Mariculture on Total and Active Bacterial Communities in the Core Sediments of the Ailian Bay, North China. Sci. Total Environ..

[68] Priyadarshanee M., Das S. (2021). Biosorption and Removal of Toxic Heavy Metals by Metal Tolerating Bacteria for Bioremediation of Metal Contamination: A Comprehensive Review. J. Environ. Chem. Eng..

[69] Dhanji-Rapkova M., Teixeira Alves M., Triñanes J.A., Martinez-Urtaza J., Haverson D., Bradley K., Baker-Austin C., Huggett J.F., Stewart G., Ritchie J.M. (2023). Sea temperature influences accumulation of tetrodotoxin in british bivalve shellfish. Sci. Total Environ..

[70] Banker R.M.W., Lipovac J., Stachowicz J.J., Gold D.A. (2022). Sodium Molybdate Does Not Inhibit Sulfate-Reducing Bacteria but Increases Shell Growth in the Pacific Oyster Magallana Gigas. PLoS ONE.

[71] Filippini G., Dafforn K.A., Bugnot A.B. (2023). Shellfish as a Bioremediation Tool: A Review and Meta-Analysis. Environ. Pollut..

[72] Yamamoto T., Nakahara S., Hiraoka K., Fukuoka K. (2023). Efficacy of the Application of Organic Fertilizer to Oyster Growth. Mar. Pollut. Bull..

[73] Ray N.E., Li J., Kangas P.C., Terlizzi D.E. (2015). Water Quality Upstream and Downstream of a Commercial Oyster Aquaculture Facility in Chesapeake Bay, USA. Aquacult Eng..

[74] Mara P., Edgcomb V.P., Sehein T.R., Beaudoin D., Martinsen C., Lovely C., Belcher B., Cox R., Curran M., Farnan C. (2021). Comparison of Oyster Aquaculture Methods and Their Potential to Enhance Microbial Nitrogen Removal from Coastal Ecosystems. Front. Mar. Sci..

[75] Wang W., Liu C., Cui Q., Xiang C., Li S., Huang J., Negahdary M., Wan Y. (2024). Spatial and Temporal Variation of Microbial Populations and Microbial Metabolic Potential in a Tropical Marine Cage-Culture Sediment System. Ecol. Indic..

[76] Labrie M.S., Sundermeyer M.A., Howes B.L. (2023). Quantifying the Effects of Floating Oyster Aquaculture on Nitrogen Cycling in a Temperate Coastal Embayment. Estuar. Coast..

[77] Muñoz C., Hidalgo C., Zapata M., Jeison D., Riquelme C., Rivas M. (2014). Use of Cellulolytic Marine Bacteria for Enzymatic Pretreatment in Microalgal Biogas Production. Appl. Environ. Microbiol..

