# Supplementary material for: Oyster Aquaculture Impacts on Environment and Microbial Taxa in Dapeng Cove
Source: Microorganisms. 2025 Oct 30;13(11):2480. doi: 10.3390/microorganisms13112480 (PMC12654026; doi:10.3390/microorganisms13112480)
Supplement: Supplementary file 1 [file microorganisms-13-02480-s001.zip › microorganisms-3856986-supplementary.pdf]

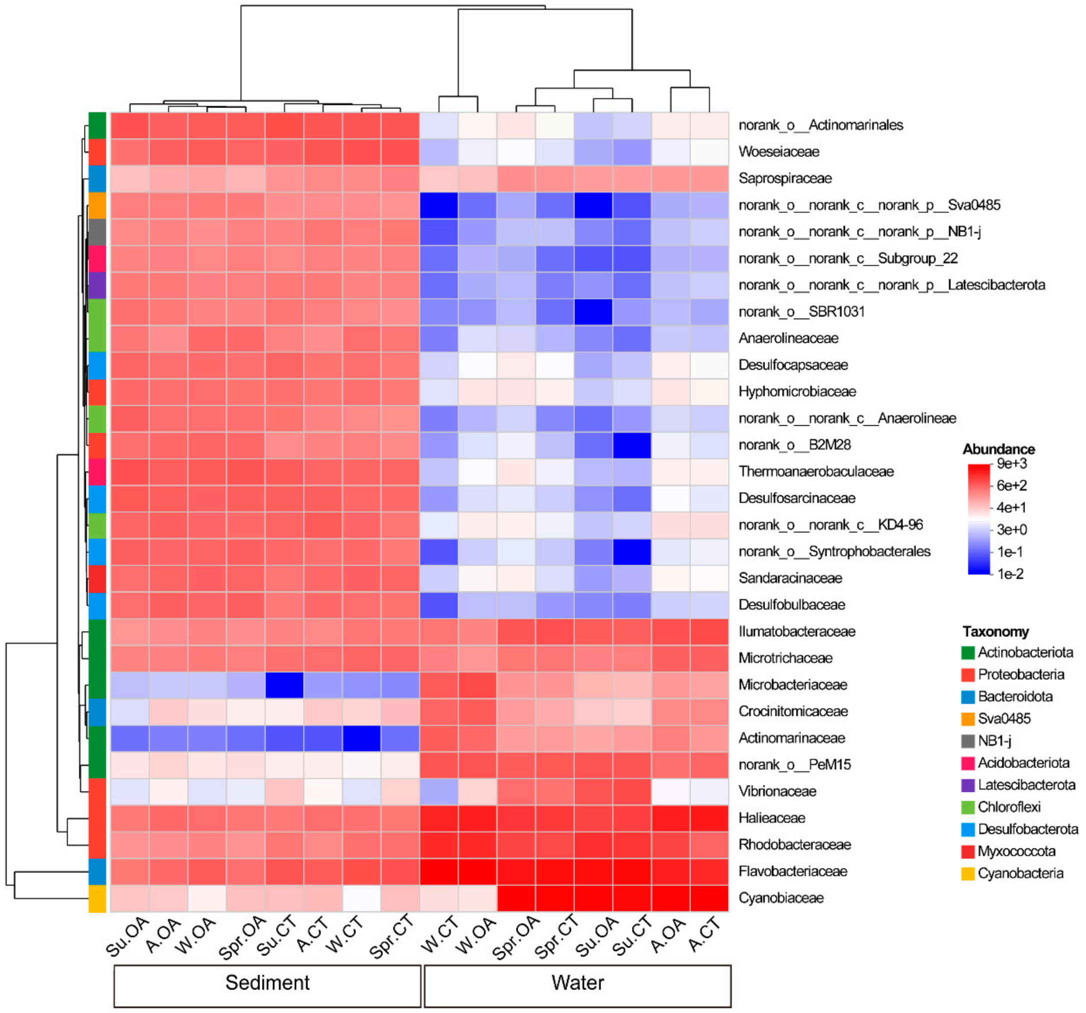

Figure S1. Hierarchical clustering heatmap of bacterial community compositions between oyster aquaculture and control areas across four seaons (Family level). (Spr.:Spring; Su.:Summer; A.:Autumn; W.:Winter.)

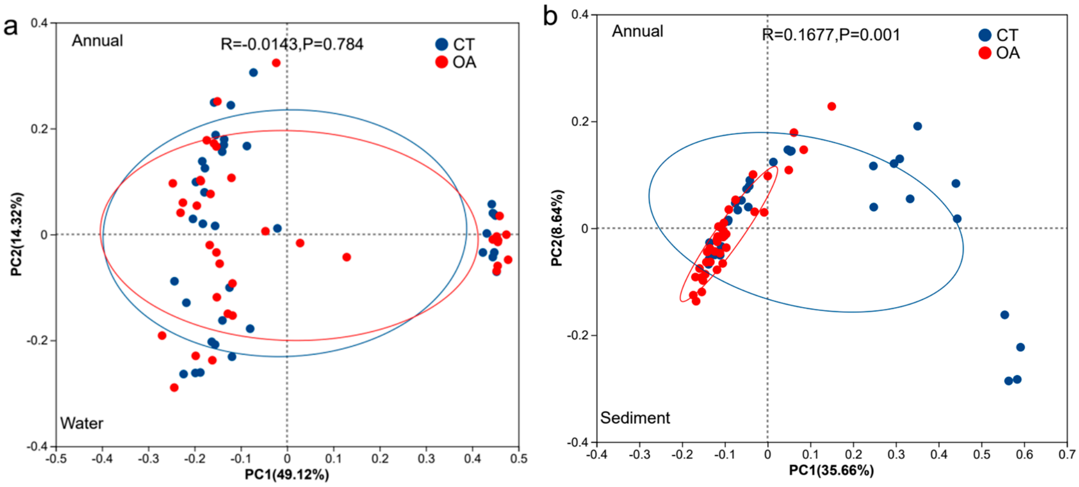

Figure S2. PCoA analysis of the bacterial community between the oyster aquaculture and control areas

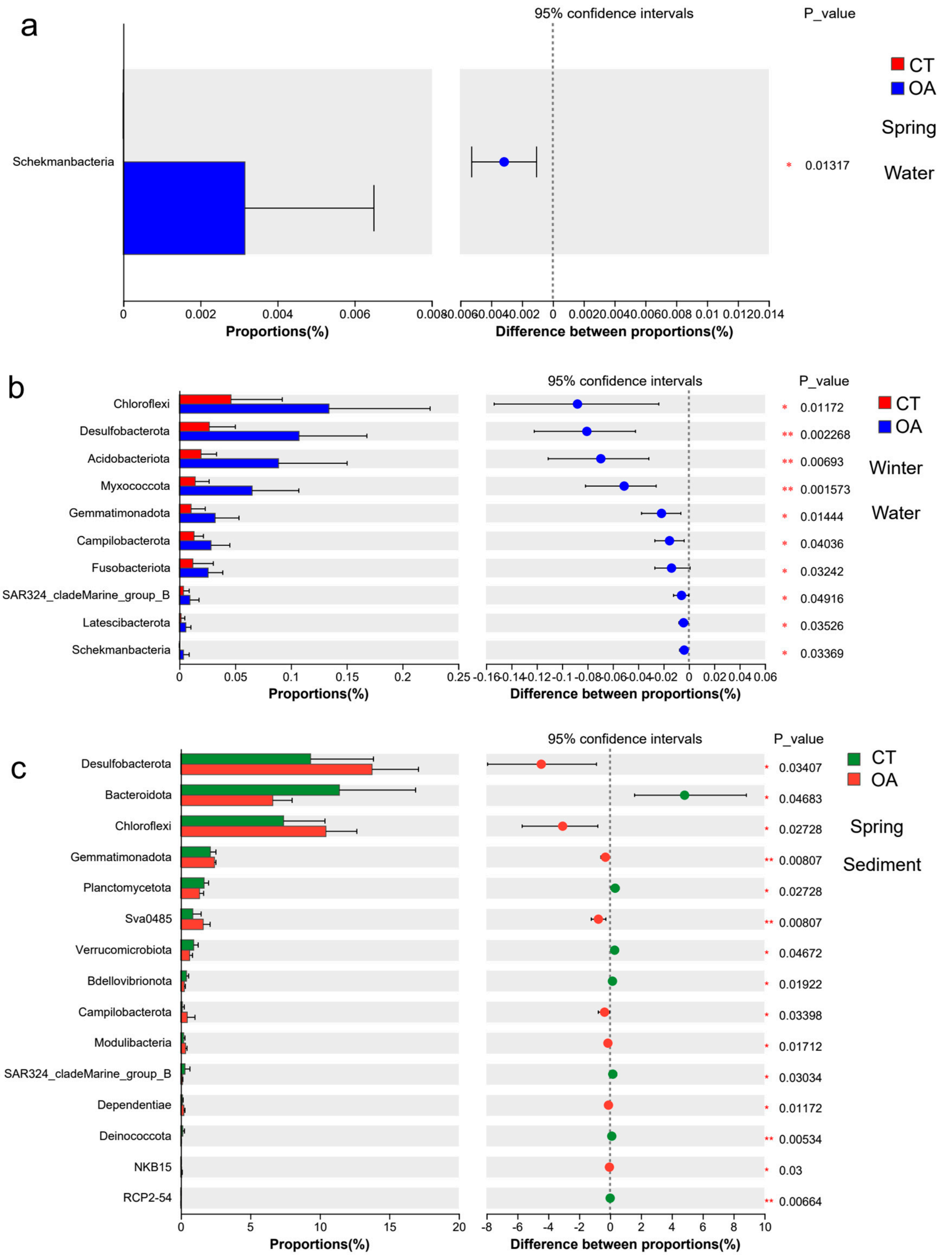
a
95% confidence intervals
P_value
CT
OA
Spring
Water
Schekmanbacteria
* 0.01317
0
0.002
0.004
0.006
0.008
Proportions(%)
Difference between proportions(%)
b
95% confidence intervals
P_value
CT
OA
Winter
Water
Chloroflexi
Desulfobacterota
Acidobacteriota
Myxococcota
Gemmatimonadota
Campilobacterota
Fusobacteriota
SAR324_cladeMarine_group_B
Latescibacterota
Schekmanbacteria
* 0.01172
** 0.002268
** 0.00693
** 0.001573
* 0.01444
* 0.04036
* 0.03242
* 0.04916
* 0.03526
* 0.03369
0
0.05
0.1
0.15
0.2
0.25
Proportions(%)
Difference between proportions(%)
c
95% confidence intervals
P_value
CT
OA
Spring
Sediment
Desulfobacterota
Bacteroidota
Chloroflexi
Gemmatimonadota
Planctomycetota
Sva0485
Verrucomicrobiota
Bdellovibrionota
Campilobacterota
Modulibacteria
SAR324_cladeMarine_group_B
Dependentiae
Deinococcota
NKB15
RCP2-54
* 0.03407
* 0.04683
* 0.02728
** 0.00807
* 0.02728
** 0.00807
* 0.04672
* 0.01922
* 0.03398
* 0.01712
* 0.03034
* 0.01172
** 0.00534
* 0.03
** 0.00664
0
5
10
15
20
-8
-6
-4
-2
0
2
4
6
8
10
Proportions(%)
Difference between proportions(%)

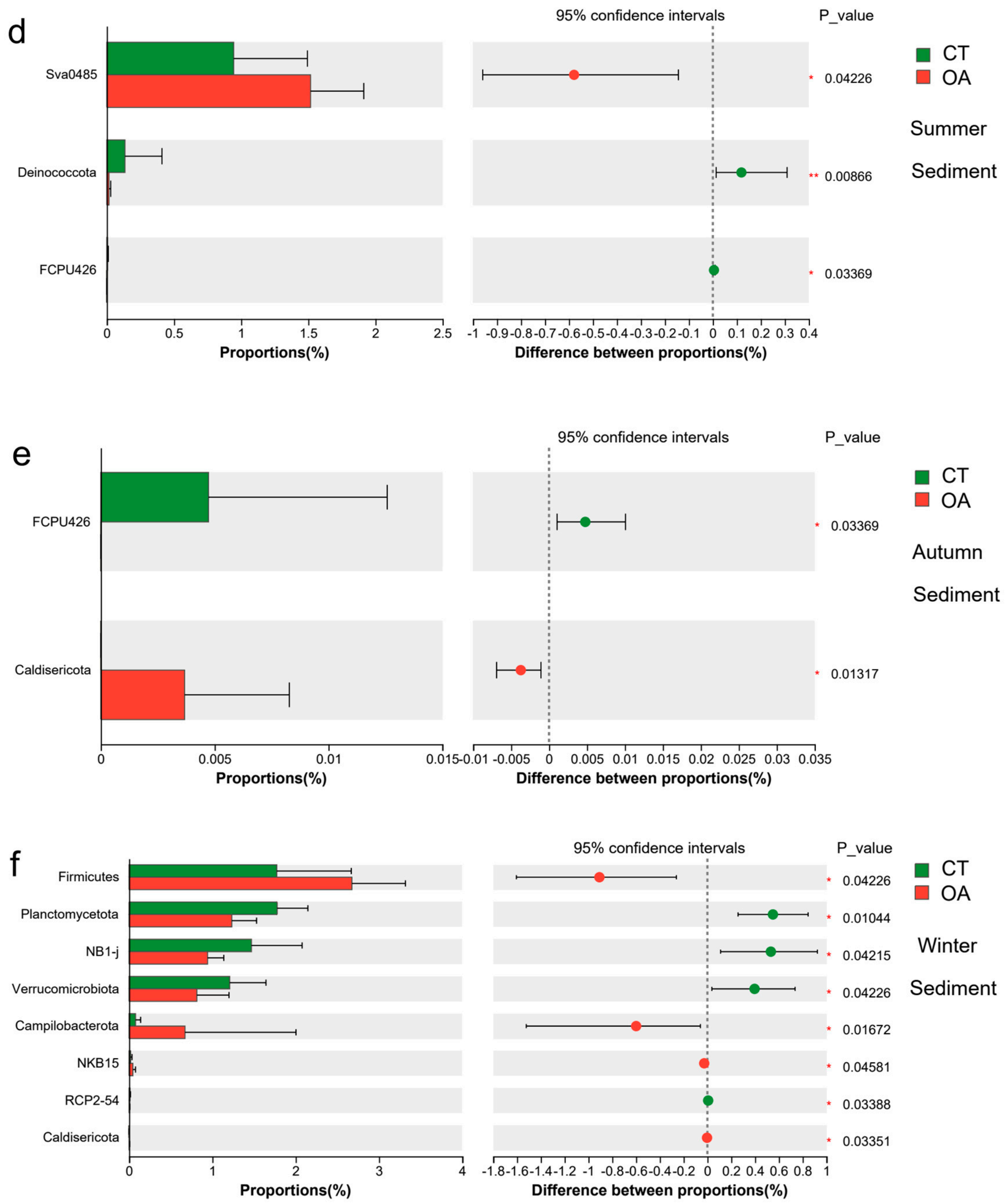

Figure S3. Bar plot for the average annual of bacterial relative abundance between oyster aquaculture and control areas

Note: * represents significant differences (*. $P < 0.05$, **. $P < 0.01$, ***. $P < 0.001$)

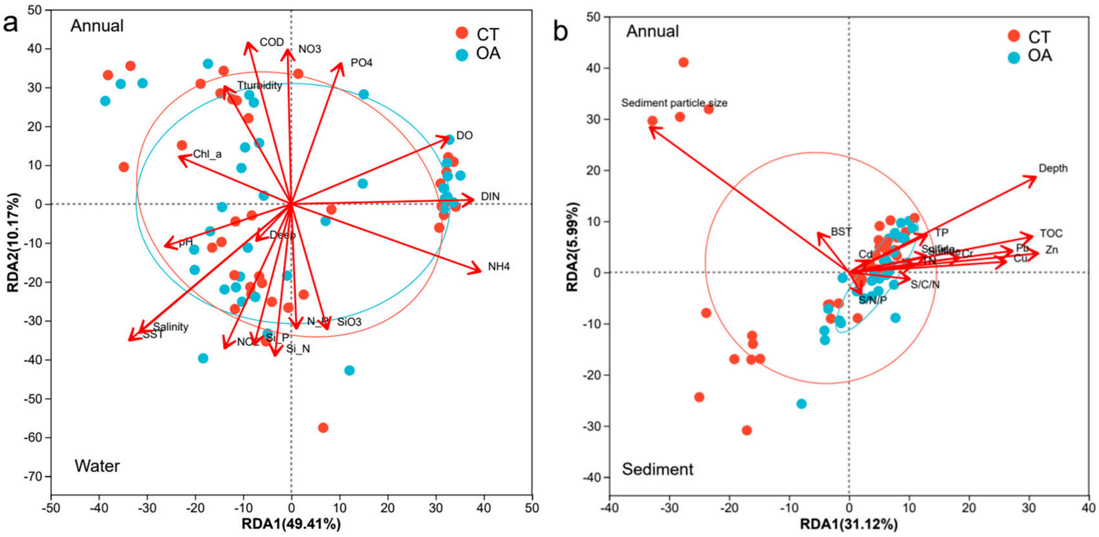

Figure S4. RDA for the oyster aquaculture and control areas bacterial communities and environmental factors

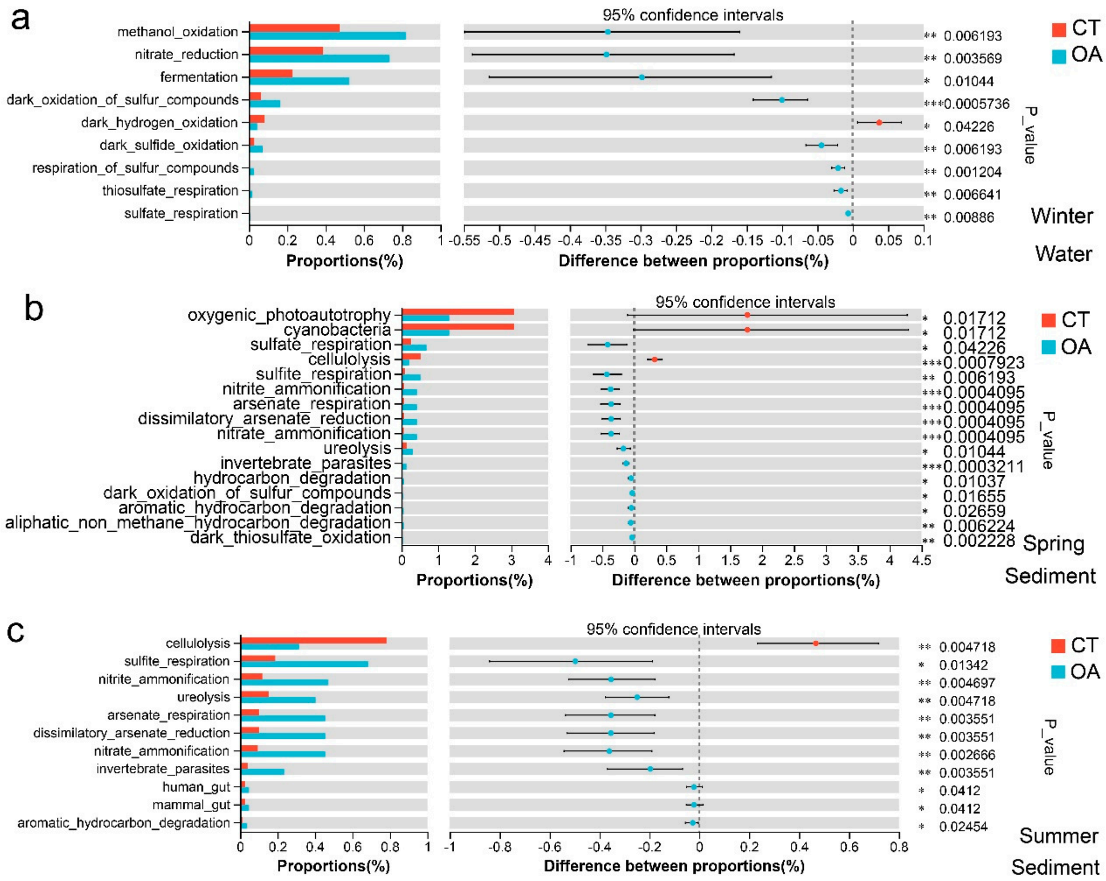

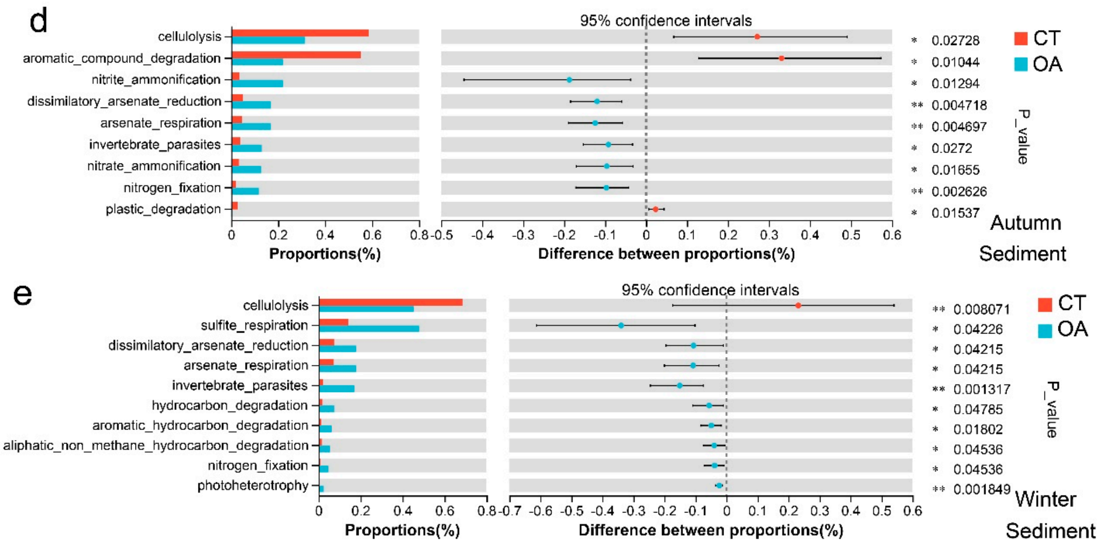

Figure S5. Analysis of potential biogeochemical functional differences in the bacterial community between oyster aquaculture and control areas.

Note: * represents significant differences (*. $P < 0.05$, **. $P < 0.01$, ***. $P < 0.001$)
